# Left ventricular function, aortic velocity, and late gadolinium enhancement assessed by real-time and single shot CMR is comparable to breath-held segmented imaging: a prospective study

**DOI:** 10.1186/1532-429X-15-S1-O51

**Published:** 2013-01-30

**Authors:** Ashish Aneja, Chikako Ono, Debbie Scandling, Ning Jin, Alice M Hinton, Michael Pennell, Subha V Raman, Orlando P Simonetti

**Affiliations:** 1 Department of Internal Medicine, Department of Cardiovascular Medicine, The Ohio State University Medical Center, Columbus, OH, USA; 2 Siemens Healthcare, Columbus, OH, USA; 3 Internal Medicine and Radiology, The Ohio State University, Columbus, OH, USA; 4 Division of Biostatistics, College of Public Health, The Ohio State University, Columbus, OH, USA; 5 Dorothy M. Davis Heart and Lung Research Institute, The Ohio State University, Columbus, OH, USA

## Background

The typical CMR exam utilizes segmented k-space acquisitions that require repeated breath-holds, a regular cardiac rhythm, and long exam times. Widespread utilization of CMR has been hampered by prolonged exam times that limit cost-effectiveness, and limited reliability in patients with irregular rhythm and/or inability to breath-hold.

## Objectives

The purpose of this study was to prospectively compare assessments of left ventricular (LV) ejection fraction (EF), aortic velocity, and late gadolinium enhancement by single-shot and real time (RT) techniques vs. traditional breath-hold segmented k-space (BH) acquisitions.

## Methods

Thirteen patients referred to the CMR lab for evaluation of undiagnosed cardiomyopathy were prospectively enrolled. All patients were in regular cardiac rhythm and able to breath-hold. Standard segmented k-space images (including cine, velocity mapping (VM), and late-gadolinium enhancement (LGE)) were acquired during breath-hold; real-time images (including cine, VM, and LGE) were acquired during free-breathing. Relevant imaging parameters are listed in Table 1. Breath-held and real-time studies for LGE were blinded and scored on a segmental basis for presence or absence of enhancement by two reviewers. LVEF was calculated using Simpson's rule on short-axis BH cines, and biplane area-length methodology on long-axis RT cines. EF results were averaged across cardiac cycles when RT cine spanned more than one heartbeat. Peak velocity through the aortic valve was assessed in BH and RT images and compared. Maximum peak velocity was used when RT images spanned more than one heartbeat.

**Table 1:** Imaging parameters for real time and breath held imaging

	Typical imaging parameters					
	Cine		Velocity mapping		Late gadolinium enhancement	

	BH	RT	BH	RT	BH	RT

Sequence Type	SSFP	SSFP	GRE	GRE-EPI	IR-GRE	IR-SSFP

TR/TE ms	2.9/1.2	2.2/1.0	5.3/2.1	10.0/7.0	8.0/4.2	2.8/1.1

Matrix	256X256	108X160	128X192	84X128	160X192	100X192

Slice Thickness (mm)	8	10	6	10	8	8

Temporal Resolution (ms)	40	60	53	40	200	180

Acceleration Rate	R2	R4	R2	R3	R1	R2

Bandwidth (Hz/pixel)	930	1488	401	2790	130	1184

Breath-hold Time (RR)	11	-	16	-	12	-

## Results

**Figure 1:**
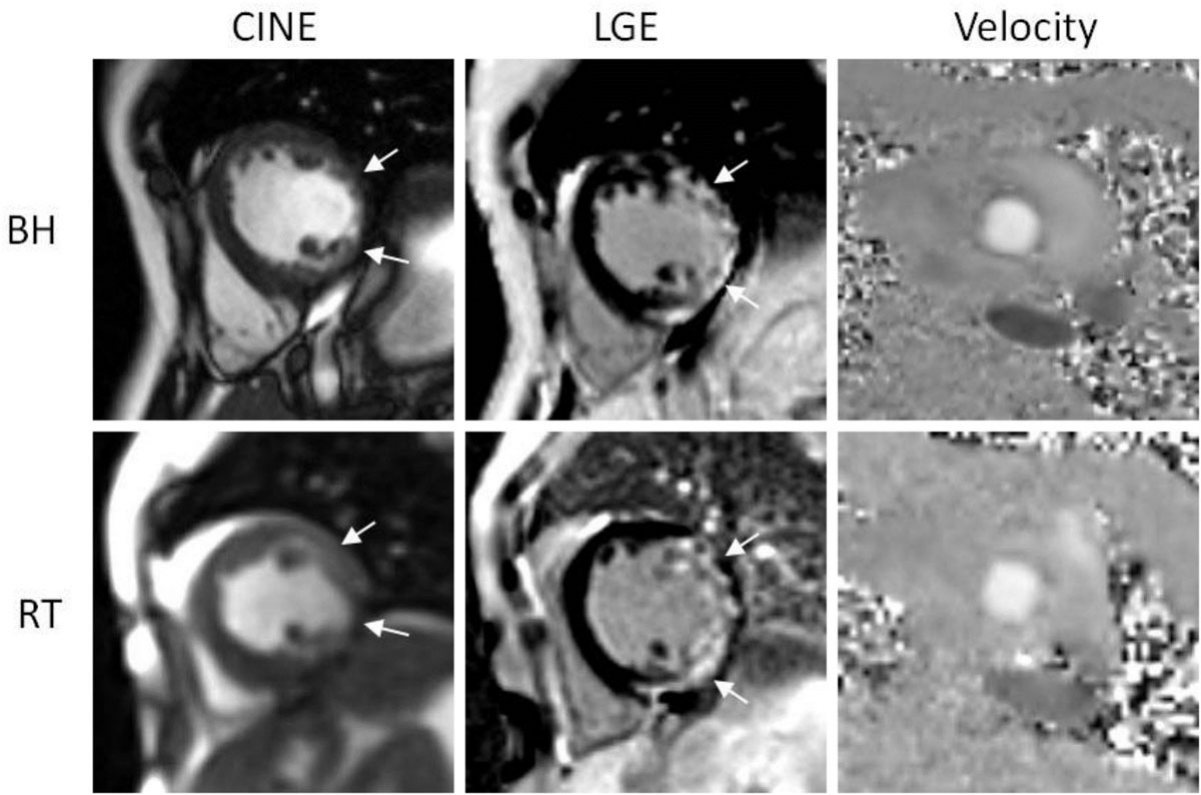
Representative example of cine, LGE and velocity breath-held and real-time imaging : arrows show areas of post-infract sub-endocardial scarring seen on real-time and breath-held images that had a corresponding lateral wall motion abnormality on both real-time and breath-held cine images.

Example images from one patient are shown in Figure 1. There was good to excellent agreement between real-time imaging and breath-held imaging for all assessed parameters: the kappa statistic for agreement for LGE was 0.73 for reviewer 1 (n=204) and 0.64 for reviewer 2 (n=202). Quantitative assessment of ejection fraction and peak aortic velocity revealed excellent concordance correlations of 0.89 and 0.91 respectively between breath-held and real-time acquisitions.

## Conclusions

Prospective, real-time data acquisitions of left ventricular ejection fraction, late-Gadolinium enhancement, and quantitative velocity mapping reveal comparable, reliable, interpretable data compared to traditional breath-held CMR methodologies. These results augur well for improved CMR throughput and reliability in the future.

## Funding

Grants from the National Institutes of Health (R01 HL102450) and Siemens Healthcare.

